# Levels and determinants of overprescribing of antibiotics in the public and private primary care sectors in South Africa

**DOI:** 10.1136/bmjgh-2023-012374

**Published:** 2023-07-31

**Authors:** Mylene Lagarde, Duane Blaauw

**Affiliations:** 1 Department of Health Policy, London School of Economics and Political Science, London, UK; 2 Center for Health Policy, University of the Witwatersrand, Johannesburg, South Africa

**Keywords:** health policy, cross-sectional survey, public health

## Abstract

Although overprovision of antibiotics in primary care is a key driver of antibiotic resistance, little is known about its determinants in low-income and middle-income countries. Patient demand and financial incentives for providers are often held responsible for overprovision. Yet, inadequate provision exists in their absence and could be fuelled by quality of care issues and incorrect beliefs of providers regarding patients’ expectations. We explored these issues in the private and public sector in South Africa, by conducting a cross-sectional study using standardised patients (SPs)—healthy individuals trained to portray a scripted clinical case to providers—presenting with symptoms of a viral respiratory infection in a sample of public and private sector clinics. We linked data from SP visits to rich survey data to compare the practices and their predictors in the two sectors. Unnecessary rates of antibiotics were similarly high in the public (78%) and private sector (67%), but private providers prescribed more antibiotics at higher risk of resistance development. In the private sector, overprescription of antibiotics diminished when consultations were more thorough, but increased for consultations scheduled later in the day, suggesting contrasting effects for provider effort and decision fatigue. We observed differences in beliefs that could be responsible for overprescription: in the public sector, a majority of providers (nurses) wrongly believed that antibiotics would help the patient recover more quickly. In the private sector, a majority of doctors thought patients would not come back if they did not receive antibiotics. Overall, this evidence suggests that different factors may be responsible for the high overprescribing rates of antibiotics in the public and private sectors. Tailored stewardship interventions are urgently needed that tackle providers’ engrained habits and incorrect beliefs.

**WHAT IS ALREADY KNOWN ON THIS TOPIC**

- Inappropriate prescribing of antibiotics in primary care is a major cause of antibiotic resistance.
- South Africa has some of the world’s highest rates of antibiotic resistance and most antibiotics are provided in primary care, a lot of them for viral respiratory infections.
- To introduce effective antibiotic stewardship programmes, governments need to know which factors drive overprescribing.

**WHAT THIS STUDY ADDS**

- We add to existing studies using standardised patients (SPs) by comparing practices in the public and private sectors.
- Private and public providers in South Africa recommended similarly high levels of unnecessary antibiotics for SPs presenting a case of viral respiratory infection.
- Private doctors recommended more antibiotics in a higher risk category for antimicrobial stewardship.
- More thorough consultations were associated with a reduction in inappropriate prescribing in the private sector, but not the public sector.
- Decision fatigue seemed to fuel unnecessary antibiotic prescribing in the private sector, with consultations occurring later in the day associated with more antibiotics.
- Public providers had incorrect beliefs about treatment effectiveness while private doctors believed most patients would want antibiotics.

**HOW THIS STUDY MIGHT AFFECT RESEARCH, PRACTICE OR POLICY**

- Interventions are urgently needed in primary care in South Africa to reduce inappropriate prescribing, both in the public and private sectors.
- Future research should aim to identify effective interventions to reduce overprescribing of antibiotics in primary care.
- Different approaches are likely to be needed in the private and public sectors.

## Introduction

Antimicrobial resistance (AMR)—the resistance developed by bacteria, viruses or fungi to existing treatments—is occurring more frequently, in more countries and for more drug classes.^1^ A recent estimate suggested that 1.27 million deaths in 2019 were directly attributable to AMR, a level of mortality equal to the combined deaths due to HIV and malaria.^2^ AMR also poses a significant threat to global health and health systems as it increases the frequency and duration of hospital stays, and treatment costs.^1^ Antibiotic resistance, a major subset of AMR, takes two forms. Individual antibiotic resistance, which may arise when individuals suffering from a particular disease (eg, tuberculosis, HIV) and develop resistance to the treatment, and public health antibiotic resistance, which is the product of misuse or overuse of antibiotics on population health, through the spread of resistant strain of bacteria within communities.^3^ Antibiotic consumption has risen globally by 65% between 2000 and 2015, mostly driven by low-income and middle-income countries (LMICs).^4^ In most health systems, the majority of antibiotics is prescribed in primary care,^5^ with large proportions of those often prescribed unnecessarily for viral respiratory tract infections (RTIs).^6–8^ Such practices have been linked to increased antibiotic resistance within communities through the spread of resistant strains of bacteria.^9^

Several factors can contribute to inappropriate antibiotic prescribing. Patients’ demand and provider financial incentives are often cited as key contributing factors. Yet, recent evidence in LMICs shows that high rates of antibiotic provision occur in the absence of patient request,^10 11^ and even when providers mention patient demand as a main factor, it is not necessarily observed in practice.^12^ In many settings, public providers have no financial incentives to recommend unnecessary drugs; in South Africa, even private providers have no profit motive to overprescribe.^13^ Two other sets of factors may play a role and have been less studied. On the one hand, overprescribing may be fuelled by quality of care shortcomings—from structural factors (eg, lack of treatment guidelines, lack of clinical training or education about AMR) to inadequate process quality of care (eg, insufficient effort of providers during consultations).^14^ On the other hand, antibiotics overprescribing may be driven by the willingness of providers to satisfy patients.^15 16^ Providers may go against their best clinical judgement and seek to satisfy what they *believe* patients expect^17 18^—a motive potentially stronger in the private sector, where providers compete for patients.

To inform the design of effective interventions, more evidence is needed in LMICs to understand the determinants of inappropriate prescribing practices by primary care providers. While these have been extensively studied in high-income settings,^19–22^ research in LMICs has been more limited. Moreover, despite the critical role played by private providers in delivering health services in LMICs, most studies have focused on the public sector. Finally, studies of prescribing decisions have usually relied on methods (eg, analysis of medical records, prescriptions or provider surveys) that suffer from several limitations.^10^ First, uncertainty about patients’ actual diagnosis due to incomplete data means that inappropriate antibiotic prescribing is prone to measurement error. Second, without knowing the attitude or requests made by patients, it is difficult to attribute to providers only the decision to treat with antibiotics. Lastly, differences in provider behaviour can be confounded with differences in patient characteristics. For example, poorer patients or patients with more complicated symptoms are likely to choose particular providers.^23^ This is a particularly relevant concern in comparative studies of the public and private sectors. To address these methodological issues, researchers have recently used standardised ‘mystery’ patients—healthy individuals sent to providers to portray specific symptoms—and shown high levels of overprovision of antibiotics.^24–29^

In this paper, we build on this recent literature to answer three questions. First, we overcome empirical challenges to obtain objective and reliable measures of antibiotic overprescription for viral respiratory illnesses in South Africa, a country that has some of the world’s highest rates of antibiotic resistance from both Gram-positive and Gram-negative bacteria.^30^ Second, we compare the rates of inappropriate prescribing in the public and private sectors. Finally, we collect detailed provider-level and consultation-level data to explore the drivers of overprovision of antibiotics, specifically seeking to tease out the role of quality of care issues from providers’ beliefs about patients’ expectations.

## Methods

### Study setting

South Africa is a middle-income country, where three-quarters of antibiotics use is in humans.^30^ Estimates suggest that 75%–80% of these antibiotics are provided at the primary care level,^31^ and most of it is unnecessary.^32^ South Africa has a comprehensive and effective legal framework ensuring that only formally trained providers can prescribe or dispense medicines. Standard treatment guidelines are well developed, and neither public nor private providers profit financially from dispensing drugs.^13^

The country is characterised by a dual healthcare system where the private and public sector offer contrasting settings. In the public sector, used by 85% of the population, primary care consultations are mostly done by nurses who typically see large numbers of patients in underfunded facilities.^33^ As part of the consultation, patients receive drugs free of charge. Providers in public primary care clinics (nurse or doctors) can only dispense drugs that are on the official primary care Essential Drugs List.^34^

Meanwhile, in the private sector, predominantly found in urban areas, primary care services are provided by medical doctors who compete for the wealthy minority. About 40% of doctors are dispensing general practitioners (GPs) who procure cheap and generic drugs which they dispense to patients as part of a flat-fee consultation; if needed, they can also write a prescription for uncommon or expensive drugs. The other 60% of doctors are prescribing GPs who write a prescription that must be filled by patients at a private pharmacy. More details about the difference between prescribing and dispensing GPs and their practices can be found in a companion study.^13^

Given this setting, it is unclear whether one should expect a higher rate of unnecessary prescriptions in the public or private sector. If quality or training issues are a key driving factor of inappropriate prescribing, one would expect higher rates of unnecessary antibiotics in the public sector. On the other hand, private providers could be particularly sensitive to (perceived) patient demand, which could fuel inappropriate antibiotic provision in the private sector.

### Study design

To compare prescribing practices in the private and public sectors, our study took place in the city of Johannesburg, the largest city in South Africa. This dual offer is typical of the healthcare system in similar large urban areas in the country (Cape Town, Durban, Braamfontein, etc) but not so much of more rural areas, where the private sector is small or inexistent.

To overcome the methodological challenges faced by other studies, we used standardised patients (SPs), a robust methodological approach that has been increasingly used to measure quality of care and overprovision in LMICs.^35^ SPs are individuals trained to pass as real patients, unbeknownst to the provider, and presenting a scripted case with prespecified symptoms and history.^35^ This method allows us to eliminate uncertainty about the patients’ attitude or actual diagnosis, and hold patient characteristics constant across all providers.

Our aim was to assess the drug dispensing and prescribing practices of primary care providers for young and healthy SPs presenting with viral bronchitis, a typical case of viral RTI.^36 37^ This choice was driven by several reasons. First, although international and national guidelines on the management of viral bronchitis^38–40^ agree that it is a self-limiting condition and that treatment should not include antibiotics, the literature suggests that it is a prime candidate for unnecessary prescribing of antibiotics,^36 37^ including in South Africa.^31^ Second, RTIs are one of the main reasons why individuals seek primary care in South Africa in general, and they are commonplace in winter, when we undertook the study.^31^ This contributes to the realism and external validity of our study. Finally, we chose healthy and young patients to exclude potential concerns of complications sometimes given as reasons for prescribing unnecessary antibiotics. Hence, our results are likely the lower bound estimates of inappropriate antibiotic prescribing for bronchitis.

We developed a textbook case of viral bronchitis with input from international and South African experts on acute RTIs. In their initial complaint, SPs explained that they had been suffering from a cough for the past 4–5 days, following a recent cold. Appropriate questioning and examination would reveal an absence of fever, a productive cough with clear mucus, no wheezing or shortness of breath or any other physical symptom (see online supplemental appendix table A1). This should lead providers to rule out pneumonia or other bacterial infections and conclude that the symptoms were most likely caused by a viral infection for which no antibiotics are indicated (see online supplemental appendix table A2).^38 39 41^ The fact that all SPs were healthy and aged between 20 and 25 years further ruled out concerns about clinical complications. Importantly for the study, SPs never disclosed any treatment preferences and never requested antibiotics. Hence, we can safely exclude that patient demand might be responsible for any antibiotic provided.

SPs were trained to act as normal patients and to depict emotional and physical aspects of the illness (eg, coughing, looking tired). They were also coached to refuse any invasive procedure (eg, blood test, nebulisation) if a provider wanted to do one as part of the consultation—but this never occurred. Immediately after the consultation, SPs recorded details of their interaction with providers during the consultation (questions asked, physical examinations done) using a prespecified checklist validated with experts (see online supplemental appendix table A3).

The SP visits occurred between 20 June and 21 August 2018. In the weeks after all SPs had completed their visits, we phoned all providers to ask if they had suspected any patient. In the private sector, where SPs were trained to capture the name of the provider seen, we phoned them directly for a debriefing. Because identification of the names of providers was more difficult in the public sector, we obtained staffing lists to cross-check it against any information collected by SPs (gender, names if possible, type of providers). When a provider claimed they had detected a ‘fake’ patient, we asked for details about patient symptoms and visit dates. At the end of the call, we invited providers to take part in a 45 min face-to-face survey.

These interviews took place between 19 November 2018 and 4 February 2019. As part of this survey, we collected background information on providers and, using a clinical vignette of the case portrayed by SPs, we asked providers the likelihood (on a scale of 0–100) that (i) the most likely cause of the patient’s illness was viral; (ii) antibiotics would help the patient recover more quickly and (iii) the patient would come back to see them if they did not receive antibiotics. Finally, we tested providers’ knowledge of AMR with a simple quiz.

### Study sample

Assuming a 5% significance level, a power of 80% and a level of inappropriate prescribing in the public sector of 85% (obtained in a small pilot), we estimated that 100 patient-provider interactions in each sector would allow us to detect a difference by 16.6 percentage points.

Our sample was chosen to be typical of the primary care system in Johannesburg. In the private sector, where primary care services are almost exclusively offered by dispensing and prescribing doctors (with a handful of pharmacies and nurse clinics also offering consultations). For our sample, we contacted all GP practices included in a commercial database covering 80% of private providers in Johannesburg. We selected a proportional-to-size random sample of 100 consenting dispensing and prescribing GPs (see details in online supplemental figures A1-A2.

10.1136/bmjgh-2023-012374.supp1 Supplementary data

In the public sector, primary care is provided mostly by municipality clinics, as well as a few larger community health clinics that also provide specialist services. Our public sector sample included all of the 73 local municipality clinics functioning at the time of the study in Johannesburg. To achieve our target of 100 visits, we planned 2 visits in the larger clinics of the sample.

### Analysis

The name and class of drugs dispensed and prescriptions given to patients were systematically recorded and coded with the help of pharmacists. The main outcome of the analysis was whether the treatment included antibiotics. We also analysed the type of antibiotics, coding the active ingredient and whether it was on the Access, Watch or Reserve lists according to the 2021 AWaRe classification by WHO, which aims to promote appropriate antibiotic use.^42^ ‘Access’ antibiotics are widely available and should be used as the first choice for most common infections; ‘Watch’ antibiotics are more potent and should be used with caution to preserve their effectiveness; ‘Reserve’ antibiotics are the last resort options and should be used only when other drugs are ineffective or unavailable due to resistance or toxicity concerns.

We used data from the SP debriefing questionnaire to construct two measures of process quality of care during the consultation: duration of the consultation, and whether a provider had retrieved the three most discriminating pieces of information that would help them rule out that the cause of the symptoms was bacterial and therefore the need for antibiotics. These three elements were: the absence of fever (discovered either by asking the patient or measuring their temperature); clear lungs with no sign of pneumonia and a clear throat with no sign of inflammation (both discovered if examined). Next, we constructed two measures of provider fatigue. First, we used the number of patients waiting to be seen when the SP arrived as a proxy of patient workload. Second, given that all providers see patients all day, we used the median time of consultation to determine whether a consultation occurred early or late in the day.

Our unit of analysis was a provider-patient interaction. Using data from the SP questionnaires and provider survey, we ran logistic regressions to explore the correlates of prescribing (i) any antibiotic and (ii) any antibiotic on the Watch or Reserve list. Regressions including provider data could only be run in the subsample of consultations done by providers completed the follow-up questionnaire (ie, the ‘interview’ sample). We analysed all data with Stata V.17.

### Ethics

In the public sector, facility managers consented to the unannounced SP visits while consent for the detection and face-to-face surveys was obtained from individual providers. In the private sector, doctors consented to receive unannounced SPs and to participate the detection and face-to-face knowledge surveys. All study participants were assured of confidentiality of the study, since only aggregate-level outcomes would be reported.

### Patient and public involvement

The study design was informed by prior evidence describing concerns with quality of care and overprescription of antibiotics. Because study participants were medical practitioners, no patients were involved in study recruitment or conduct. Participating providers were invited to dissemination events where aggregate study results were presented and discussed and received a policy brief describing the study results electronically.

## Results

Table 1 describes the main characteristics of facilities and providers in the public and private sectors. Several differences stand out, echoing the dual system in South Africa previously mentioned. First, private facilities were more likely to be located in wealthier areas, while public ones were more evenly distributed. Second, providers in the private sectors were exclusively medical doctors while 95% of public providers were nurses. Third, the workload of providers was markedly higher in the public sector, with 82 patients seen on average the previous week, against 22 for private doctors—these differences translated in much longer waiting times in the public sector.

**Table 1:** Characteristics of providers

	All providers	Public sector providers	Private sector providers	P value*
Panel A: facility sample				
Average no of patients waiting	7.77 (8.58)	14.57 (8.87)	2.81 (3.41)	0.000
Average waiting time (min)	99.60 (89.06)	176.15 (72.90)	43.71 (49.38)	<0.001
Location				
Located in Q1 (poorest)	0.12 (0.32)	0.21 (0.41)	0.05 (0.22)	<0.001
Located in Q2 ward	0.14 (0.35)	0.18 (0.39)	0.12 (0.33)	
Located in Q3 ward	0.12 (0.32)	0.15 (0.36)	0.09 (0.29)	
Located in Q4 ward	0.28 (0.45)	0.29 (0.46)	0.27 (0.45)	
Located in Q5 (richest)	0.35 (0.48)	0.18 (0.39)	0.47 (0.50)	
N (facilities)	173	73	100	
Panel B: interviewed provider sample				
Location				
Located in Q1 ward (poorest)	0.12 (0.33)	0.26 (0.44)	0.05 (0.22)	0.002
Located in Q2 ward	0.18 (0.38)	0.23 (0.43)	0.15 (0.36)	
Located in Q3 ward	0.06 (0.25)	0.07 (0.26)	0.06 (0.24)	
Located in Q4 ward	0.28 (0.45)	0.26 (0.44)	0.29 (0.46)	
Located in Q5 ward (richest)	0.36 (0.48)	0.19 (0.39)	0.45 (0.50)	
Male	0.48 (0.50)	0.19 (0.39)	0.63 (0.48)	<0.001
Age	51.83 (12.60)	48.44 (12.84)	53.61 (12.18)	0.029
Ethnicity				
Black or coloured	0.50 (0.50)	1.00 (0.00)	0.24 (0.43)	<0.001
White	0.31 (0.47)	0.00 (0.00)	0.48 (0.50)	
Indian/Other	0.18 (0.39)	0.00 (0.00)	0.28 (0.45)	
Doctor	0.67 (0.47)	0.05 (0.21)	1.00 (0.00)	<0.001
No of patients (previous week)	42.84 (123.51)	82.56 (205.46)	22.01 (13.69)	0.009
N (individual providers)	125	43	82	

For the facility sample data, on average waiting time is based on information collected by SPs. We used the Gauteng city region quality of life survey to construct socio-economic quintiles of local areas (ward), and allocated each provider to a quintile according to their location. Additional data in the sample of interviewed provider (panel B) come from provider interviews undertaken as part of the study with the subset of doctors who agreed to the interview, after all SPs had completed their visits.

*The p value is based on t-test for means and χ^2^ tests for proportions comparing the private and public sector characteristics.

SP, standardised patient.

We completed a total of 201 SP visits, 102 in the public sector and 99 in the private sector. Only three SPs were detected, all in the private sector. Table 2 describes the treatment recommended to SPs and some measures of process quality of care during the consultation. Overall, antibiotics were recommended in about 73% of consultations, with a slightly lower proportion in the private sector (67%), though not statistically different from that in the public sector (78%). The majority of antibiotics were on the Access list, although antibiotics on the Watch list were recommended in 12% of consultations, a pattern mostly driven by private providers who recommended these drugs in 20% of consultations (against 5% for public providers, p<0.001). SPs received many other unnecessary medicines, again with differentiated prescribing patterns across sectors. In the private sector, steroids were given in 50% of consultations and bronchodilators in 28% (against respectively 3% and 1% for public providers). Meanwhile, antihistamines were recommended in 41% of public sector consultations, against 18% in the private sector.

**Table 2:** Descriptive results: prescribing decisions and process quality of care

	All consultations	Public sector consultations	Private sector consultations	P value*
Patient treatment includes				
Number of drugs	2.84 (2.67 to 3.00)	2.30 (2.12 to 2.49)	3.38 (3.16 to 3.61)	<0.001
Antibiotics	72.6% (65.9 to 78.7)	78.4% (69.2 to 86.0)	66.7% (56.5 to 75.8)	0.061
‘Access’ antibiotics	60.7% (53.6 to 67.5)	74.5% (64.9 to 82.6)	46.5% (36.4 to 56.8)	<0.001
‘Watch’ antibiotics	12.4% (08.2 to 17.8)	4.9% (01.6 to 11.1)	20.2% (12.8 to 29.5)	0.001
Steroids	26.4% (20.4 to 33.0)	2.9% (00.6 to 08.4)	50.5% (40.3 to 60.7)	<0.001
Bronchodilators	14.4% (09.9 to 20.1)	1.0% (00.0 to 05.3)	28.3% (19.7 to 38.2)	<0.001
Antihistamines	29.9% (23.6 to 36.7)	41.2% (31.5 to 51.4)	18.2% (11.1 to 27.2)	<0.001
Process quality of care measures				
Consultation duration (min)	8.91 (8.29 to 9.52)	7.77 (7.01 to 8.54)	10.07 (9.14 to 11.00)	<0.001
Number of essential questions asked	7.37 (6.85 to 7.90)	6.13 (5.41 to 6.84)	8.66 (7.95 to 9.36)	<0.001
Number of essential examinations done	3.93 (3.61 to 4.24)	2.60 (2.30 to 2.90)	5.29 (4.88 to 5.70)	<0.001
Provider retrieved discriminating information	32.8% (26.4 to 39.8)	5.9% (02.2 to 12.4)	60.6% (50.3 to 70.3)	<0.001
Number of observations	201	102	99	

Numbers indicate mean or proportions, with 95% CIs in parentheses. Sample sizes are numbers of standardised patient consultations.

*The p value is based on t-test for means and χ^2^ tests for proportions comparing the private and public sector characteristics.

Turning to measures of care quality, we found that consultations lasted on average 9 min, with providers asking on average about seven questions and undertaking almost four physical examination. Process quality of care in the private sector was found to be higher than in the public sector, as suggested by significant differences in the duration of consultations, the number of questions asked and examinations done. Focusing on three key essential pieces of information that could help a provider exclude a bacterial infection (absence of fever, clear throat, clear lungs), we found stark differences across sectors. The necessary examinations (or questions) to obtain these elements were only done in 6% of consultations in the public sector, against 61% for the private sector. This difference likely stems from the difference in provider clinical skills in the two sectors, as doctors are systematically trained to follow a differential diagnosis process, while nurses are not.

**Figure 1:**
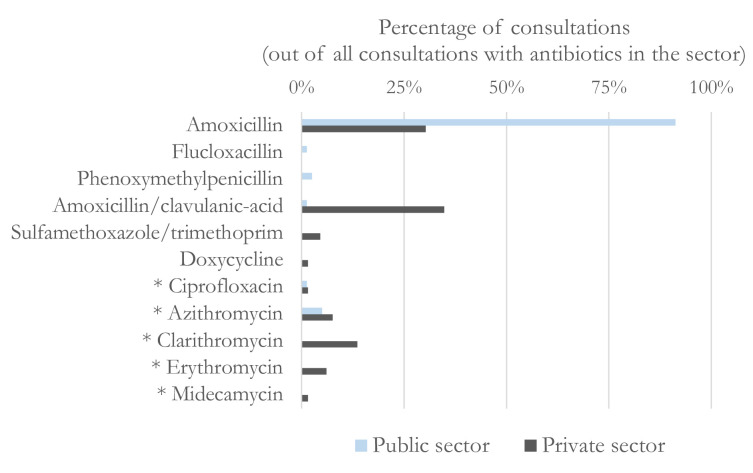
Choice of type of antibiotics, by sector. *Active ingredients on the WATCH list of the WHO, due to resistance and toxicity concerns.

Providers from the public and private sectors recommended different types of antibiotics (figure 1). While 91% of patients who received antibiotics in the public sector were given amoxicillin, the selection was more varied in the private sector, with 30% receiving amoxicillin, 35% amoxicillin combined with clavulanic acid and 14% clarithromycin, an antibiotic on the Watch list. A similar analysis separating the choices of dispensing and describing GPs (shown in online supplemental appendix figure A3) confirms the public-private difference in antibiotic choices, and highlights some heterogeneity within the private sector, as dispensing doctors’ choices appear closer to those of public sector providers. For example, 52% of antibiotics dispensed by dispensing GPs were amoxicillin, against 14% for prescribing ones. Dispensing doctors only chose an antibiotic on the Watch list in 16% of antibiotic treatments, against 44% for prescribing doctors.

The differences in antibiotic prescribing between the public and private sectors are not explained by the type of drugs public sector nurses and private doctors were able to prescribe. Indeed, all but two of the antibiotics chosen by private doctors (erythromycin and midecamycin) were on the Primary Healthcare Standard Treatment Guideline and Essential Medicine List,^43^ meaning that the other 13 antibiotics were available in all public clinics and that nurses could prescribe them.^34^

A formal analysis (shown in online supplemental appendix table A4) confirms that patients in the private sector were not less likely to receive antibiotics than in the public sector (OR 0.60; p=0.267), perhaps because our study was underpowered to detect this difference in prescribing levels at standard levels. However, private sector providers were much more likely to prescribe an antibiotic on the Watch list than public sector ones (OR 4.81; p=0.021).

**Figure 2:**
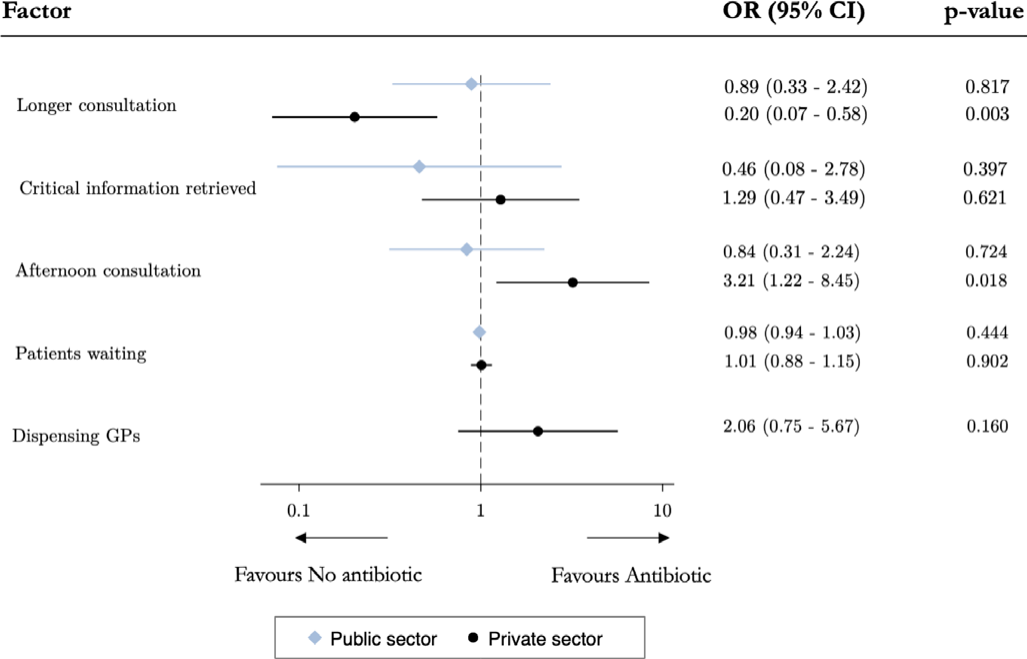
Factors associated with antibiotic prescribing, by sector. GP, general practitioner.

Given the differences in the characteristics of private and public providers highlighted in table 2, we study the factors associated with antibiotic prescribing separately for the two sectors. Figure 2 presents the correlates of prescribing any antibiotics. In the public sector, none of the measures of higher care quality during the consultation (longer consultation or whether the provider had retrieved three key elements of information to rule out bacterial information) or provider fatigue (workload or time of the day at which the consultation occurred) were predictive of antibiotic prescribing. By contrast, in the private sector, longer consultations were significantly associated with a reduced likelihood of recommending antibiotics (OR 0.20; p=0.003) and consultations that occurred later in the day were associated with more antibiotics prescribing (OR 3.21; p=0.018).

Table 3 presents the results from a survey with the 125 providers who could be identified and agreed to the face-to-face interview (see online supplemental appendix for differences between the main sample and interview sample). Mirroring the differences in the type of public and private providers (ie, public nurses vs private doctors), there are some sharp differences in their knowledge and beliefs related to the case portrayed by SPs. Overall, 84% of providers knew that the SP case was likely due to a viral infection, a proportion similar in the public and private sectors (respectively 77% and 88%, p=0.109). However, only 58% of providers believed that antibiotics would not help the patient recover more quickly, a proportion significantly higher among private providers (68% vs 40% of public providers, p=0.002). Beliefs about the impact of refusing antibiotics to patients also differed markedly by sector; 72% of private doctors thought the patient would not come back if no antibiotics was prescribed, against only 47% for public providers (p=0.008). Lastly, results showed a lower awareness of AMR among public providers compared with private ones (2.28 vs 3.46, p<0.001). These results should be taken with some caution given the attrition for the provider survey. A sensitivity analysis shown in online supplemental appendix table A5 suggests that those differences hold if providers who did not take part in the follow-up surveys had low knowledge in both sectors, but not if they had high knowledge.

**Table 3:** Knowledge of providers

	All providers	Public providers	Private providers	P value*
Knows cause of illness is viral	0.84 (0.37)	0.77 (0.43)	0.88 (0.33)	0.109
Thinks antibiotics unlikely to help patient recover	0.58 (0.49)	0.40 (0.49)	0.68 (0.47)	0.002
Believes patient will not come back if no antibiotics	0.56 (0.49)	0.47 (0.50)	0.72 (0.45)	0.008
AMR knowledge score (out of 5)	3.06 (1.22)	2.28 (1.18)	3.46 (1.03)	<0.001
Number of observations	125	43	82	

Numbers show mean or proportion with SD in parentheses. For the facility sample data, on average waiting time is based on information collected by SPs. We used the Gauteng city region quality of life survey to construct socio-economic quintiles of local areas (ward), and allocated each provider to a quintile according to their location. Additional data in the sample of interviewed provider (panel B) come from provider interviews undertaken as part of the study with the subset of doctors who agreed to the interview, after all SPs had completed their visits.

*The p value is based on t-test for means and χ^2^ tests for proportions comparing the private and public sector characteristics.

AMR, antimicrobial resistance; SP, standardised patient.

In additional analysis (see online supplemental appendix tables A6 and A7), we explored the determinants of antibiotic prescribing in this interview sample. The results confirm the previous analysis in the full sample. In the public sector, there was no evidence that provider effort, fatigue or beliefs were associated with lower antibiotic prescribing. By contrast, in the private sector, longer consultations were still strong predictors of lower likelihood of inappropriate prescribing, while the fatigue effect in late consultations remained a key behavioural determinant. There was also evidence that better knowledge (especially higher awareness of AMR) had a moderating effect in private providers’ practices, while we found no such evidence in the public sector.

## Discussion

We used SPs to study the levels and determinants of overprescribing of antibiotics for a simple case of viral respiratory infection in the public and private sectors in South Africa. Our findings confirm the existence of high levels of inappropriate prescribing of antibiotics in this country, with 73% of SPs receiving an antibiotic that was unnecessary according to clinical guidelines. Such levels echo results from a few studies done in South Africa, where data from real patients using medical records,^44^ insurance claims^6^ and surveys of prescribing practices^45^ also showed low adherence to recommended clinical guidelines. The fact that <1% of providers detected SPs provide further reassurance about the validity of our results.

Although to our knowledge this was the first study to use a case of viral bronchitis, a couple of SP studies in LMICs have looked at antibiotic prescribing for other cases of respiratory viral infections (cold symptoms). Similar or higher levels of inappropriate prescribing were found, with 95% of SPs receiving antibiotics in private not-for-profit facilities in Tanzania^11^ and 69% in public hospitals in China.^46^ However, South African private doctors prescribed significantly more antibiotics from the WHO’s medium-risk Watch category compared with Tanzanian private providers—respectively 20.2% against 8%.

We found a slightly higher rate of unnecessary antibiotics prescribed in the public sector (78.4%), compared with the private sector (66.7%), but the difference was not statistically significant in our relatively small sample. However, patients seen in the private sector were significantly more likely to receive an antibiotic from the Watch list than those seen in the public sector. Still, caution should be exercised when interpreting the difference in unnecessary antibiotic prescription between sectors, given that these differences are conflated with stark differences in provider characteristics. Hence, variation in the rate or choice of antibiotics may reflect several factors. Provider training is one reason, with less qualified providers (public sector nurses) being often more likely to prescribe antibiotics.^47 48^ Differences in perceived patients’ expectations could be another reason, with private doctors—especially prescribing doctors—expecting patients to want more expensive drugs.^13^ Isolating the impact of the sector (working environment, incentives) from the provider effect would require a research design comparing the same providers observed in the two different settings.^49^ Because the dual practice arrangements required for such a study do not exist in South Africa, one cannot disentangle both effects in this setting.

Our findings confirm the complex relationship between unnecessary antibiotics prescribing and providers’ effort exerted by in the consultation, and they highlight the importance of the role of beliefs in provider decision-making. A study in China found that more effort in the consultation was associated with a reduction in antibiotic prescribing,^29^ which echoes our results in the private sector. However, our findings in the public sector concur with the lack of relationship found in Tanzania.^11^ Our study adds to this limited evidence by examining simultaneously the role of providers’ beliefs, and contrasting determinants in the private and public sectors. Although the rates of inappropriate prescribing were similar among private and public providers, our results suggest that different factors may drive these behaviours. Indicators of process quality of care were particularly low for consultations in the public sector and despite a majority of providers suspecting that the case was caused by a virus, many held incorrect beliefs about the benefits of antibiotics. While we found no evidence that specific factors predicted inappropriate prescribing, the combined high prevalence of low quality of care and incorrect beliefs among public providers point to the existence of inadequate habits and shortcuts, which act as deep-rooted obstacles preventing appropriate prescribing. Meanwhile, in the private sector, there was also suggestive evidence that inappropriate prescribing could be partly driven by providers’ widespread *beliefs* that patients want antibiotics, with the prescription of stronger antibiotics (on the WHO’s Watch list) pointing to a perception that private patients expect stronger, more expensive drugs.^13^ Previous work in high-income settings has underlined the critical role of providers’ response to perceived patient demand.^19^ More work is needed in LMICs to explore the role of such perceptions and beliefs in overprescribing.

Finally, our study points to the role of cognitive factors in inappropriate prescribing.^50^ We failed to find an association between the number of patients waiting to be seen and treatment choices, but the strongest predictor of antibiotic prescribing in the private sector was the timing of the consultation, specifically whether it occurred late in the working day of the provider. This result echoes studies from India^35^ and the USA,^51^ where these results have been likened to a fatigue effect as the cumulative cognitive demand of providers’ decisions *“may erode [their] abilities to resist making potentially inappropriate choices”*.^51^ This mechanism is also plausible in the private sector where providers receive patients throughout the day. Physical or cognitive fatigue may lead physicians to rely on simple heuristics, or default options, which require less cognitive effort than undertaking a thorough consultation to rule out several diagnostic options. We cannot pinpoint exactly which mechanism is at play here, but future research that collects granular information about provider’s activities over the day could explore this issue further.

Our study had limitations. The external validity of the results is limited by the use of a single case (a viral respiratory infection), and by the fact that the private sample was random within the 42% of doctors who agreed to take part in study. Regarding the first concern, it is useful to remember that RTIs are the most common reason for primary care consultations in South Africa, and in many other LMICs. Therefore, they represent a large volume of all primary care consultations, and a known source of inappropriate antibiotic prescribing.^31 52^ It is difficult to ascertain the effect of the limited participation rate of private doctors, which is similar to recent SP studies in the private sector in South African metropoles.^27^ If better providers were more likely to take part in our study, we may have underestimated inappropriate prescribing in the private sector. Finally, despite its advantages, the use of SPs is limited by the fact that they provide insights into providers’ behaviours with ‘new’ patients, which could differ from those they have with regular patients. This is unlikely to be a concern in the public sector, given its large patient volumes and staff turnover. It could be more relevant in the private sector, where providers could choose to overprescribe antibiotics more to new clients to increase the probability of future visits.

Our results have several implications for policy and research. First, they suggest that the prescribing decisions of primary care providers is a ﬁrst-order problem in the management of AMR in South Africa. Yet, stewardship initiatives almost exclusively focus on hospitals.^53^ Second, they show that interventions are needed at the primary care level, in the public and private sectors, to tackle providers’ incorrect beliefs and deep-seated prescribing habits. It would be important to understand how incorrect prescribing habits develop in order to pre-empt them from taking roots. More research is also needed to develop innovative solutions suitable for resource-constrained healthcare systems where effective monitoring systems are fragmented or unreliable. Low-cost behavioural interventions, consisting of feedback or nudges that slightly modify providers’ choice environment, may constitute a fruitful avenue for operational research.^16^

In conclusion, we add to the limited evidence on antibiotic abuse by private and public primary care providers in LMICs in general, and South Africa in particular. This study highlights the failure of primary care providers to act as antibiotic stewards and suggests that engrained prescribing habits and beliefs contribute to their behaviours. Our findings can help inform the design of future research and policy initiatives related to stewardship interventions in South Africa.

## Data Availability

Data are available on reasonable request.
