# Supplementary material for: Levels and determinants of overprescribing of antibiotics in the public and private primary care sectors in South Africa
Source: BMJ Glob Health. 2023 Jul 31;8(7):e012374. doi: 10.1136/bmjgh-2023-012374 (PMC10391785; doi:10.1136/bmjgh-2023-012374)
Supplement: Supplementary data [file bmjgh-2023-012374supp001.pdf]

Levels and determinants of over-prescribing of antibiotics in the public and private primary care sectors in South Africa

ONLINE APPENDIX

1. Sampling 2
   1.1. Sample size calculation 2
   1.2. Private sector sample 2
   Appendix Figure 1: Sampling flowchart (private sector) 3
   1.3. Public sector sample 4
   Appendix Figure 2: Sampling flowchart (public sector) 4
2. Standardised patient methodology 5
   2.1. Case development and script 5
   Appendix Table A1. Standardized responses to doctors' potential questions 6
   2.2. Training of standardised patients 8
   2.3. Differential diagnosis 8
   Appendix Table A2. Alternative diagnoses for the case presented 9
   2.4. Checklist 10
   Appendix Table A3. Post-consultation checklist 10
3. Construction of variables 11
   3.1. Consultation characteristics (SP data) 11
   3.2. Provider characteristics (interview data) 11
4. Additional results 13
   Appendix Figure 3: Choice of type of antibiotics, by sector and type of private doctor 13
   Appendix Table A4. Correlates of antibiotic prescribing, whole sample 14
   Appendix Table A5. Knowledge of providers – sensitivity analysis 15
   Appendix Table A6. Correlates of antibiotic prescribing, public sector 16
   Appendix Table A7. Correlates of antibiotic prescribing, private sector 17
5. Strobe Statement 18
6. Reflexivity Statement 20

1. Sampling

1.1. Sample size calculation

Given that providers in the private sectors are only medical doctors, we hypothesised that they would be less likely to prescribe unnecessary antibiotics. To compare the proportion of patients receiving antibiotics between private and public providers, assuming a 5% significance level and a power of 80%, we estimated that we would need 121 interactions in each group to detect a difference of 15 percentage points, assuming a level of inappropriate prescribing in the public sector of 85% - based on results in a small pilot study and similar SP studies in other settings. Budget constraints meant that we could only have 100 patient-provider interactions by sector, meaning that we were powered to detect a significant difference of 16.6 percentage points. We felt that this would still be a meaningful difference between the two sectors – given the high levels of antibiotics we were expecting in the public sector, anything short of such a difference would still be considered extremely high and worrisome in the private sector.

1.2. Private sector sample

To construct a sampling frame of private doctors, we used a commercial national database of practitioners,[1] which includes approximately 80 percent of all registered doctors nationally, and significantly more in urban areas. The database included the contact details of 1,017 practicing private providers in Johannesburg. To be eligible for this study, a doctor had (1) to practice general medicine and (2) to work in a private practice. Of the 1,017 listed doctors, 205 (20.2%) were not eligible to take part in the study. We called 87% of the 812 eligible doctors (*n* =705) between March and June 2018 to invite them to take part in the study. Of those 705 doctors, 16.5% (*n* = 116) could not be reached despite several attempts;[2] 26.7 percent refused to take part (*n* = 188); 17.1 percent (*n* = 121) requested further information about the study to make their decision but never responded again, and 39.7% agreed to take part (*n* = 280). From this final group, we drew a random sample of 100 providers using proportional random sampling to obtain 39% of dispensing doctors and 61% of non-dispensing doctors, the two main groups of doctors in the private sector.[3] Each provider was visited by a pair of patients, aiming for a total of 100 consultations for each group.

[1] https://www.medpages.info/sf/index.php?page=homepage

[2] Either no one responded, or the receptionist refused to pass the communication to the doctor.

[3] Dispensing doctors are licensed to dispense drugs which are included in the cost of the consultation. Non-dispensing doctors write a prescription to patients to be filled in a pharmacy.

Appendix Figure A1: Sampling flowchart (private sector)

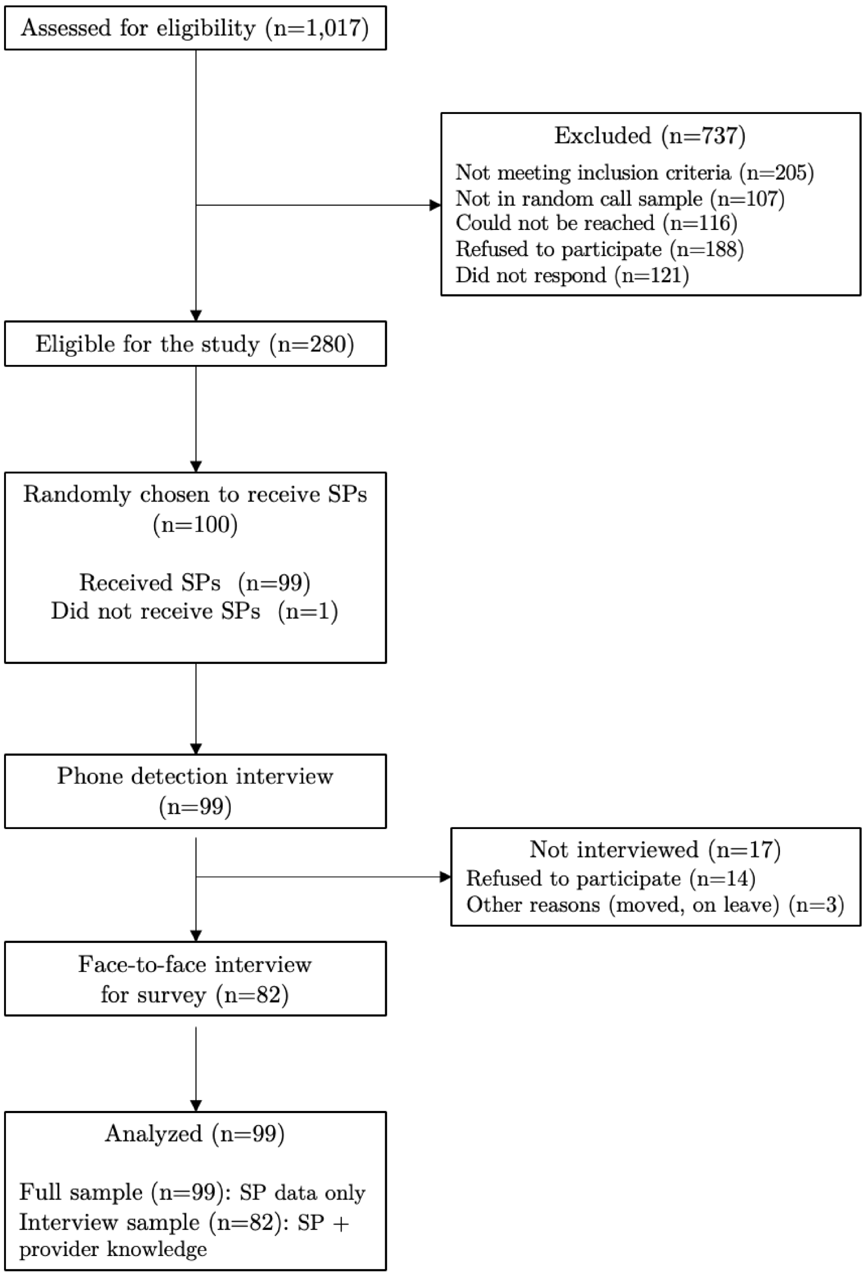

1.3. Public sector sample

In the public sector, there were only 73 primary care municipality clinics that were functioning in the Johannesburg Metro at the time of the survey. Hence we included all of them in the study, and in the largest 29 clinics we sent two patients of each type to meet the required sample size of 100 interactions in each attitudinal group, or 200 interactions in total.

Appendix Figure A2: Sampling flowchart (public sector)

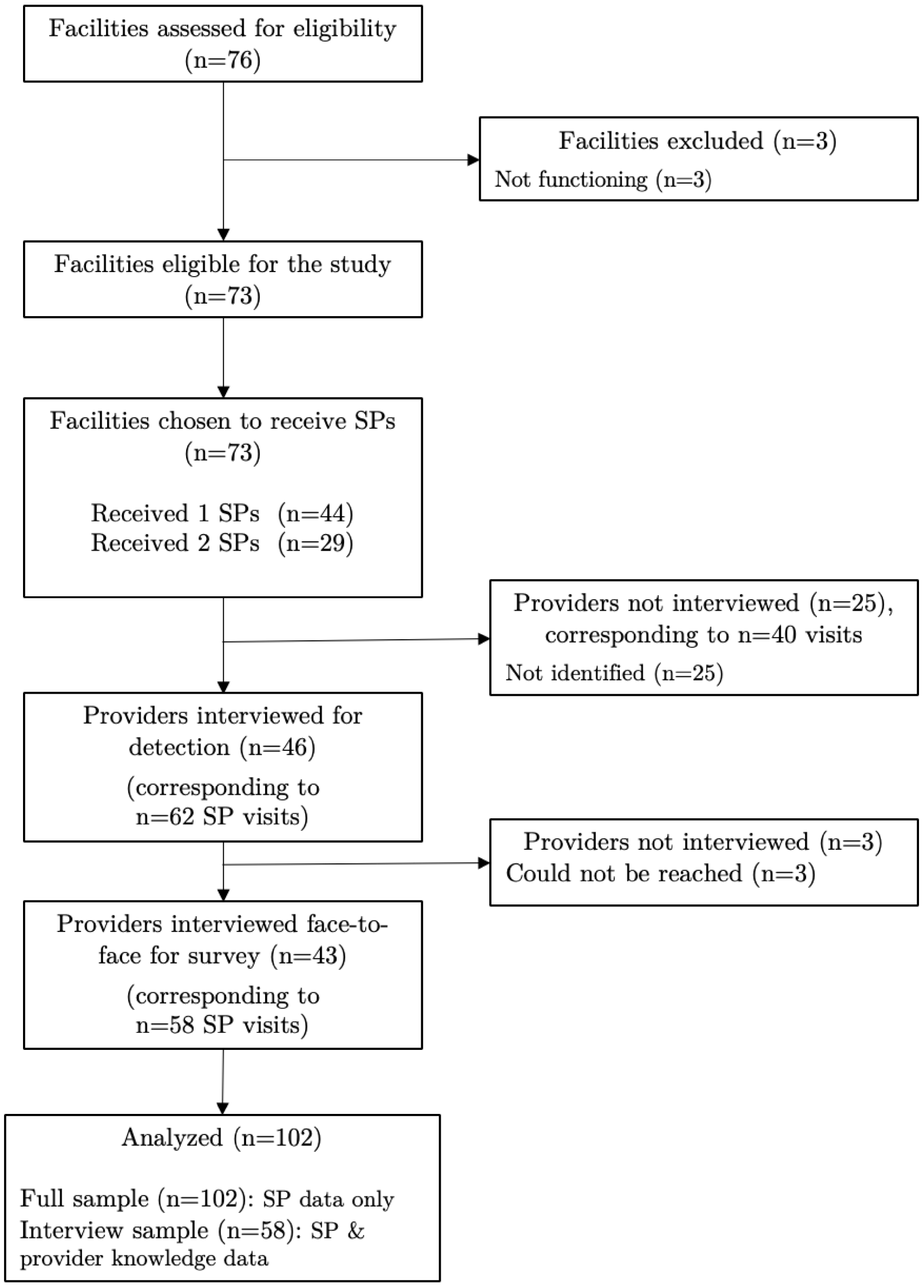

Lagarde M, Blaauw D. *BMJ Glob Health* 2023; 8:e012374. doi: 10.1136/bmjgh-2023-012374

2. Standardised patient methodology

2.1. Case development and script

Standardised patients were trained to accurately and consistently present the clinical symptoms and history of a viral respiratory infection (acute bronchitis) in a healthy adult in their early 20s. They were also tasked to take one of two potential scenarios displaying or not their treatment preference.

The case was developed in collaboration with several local medical professionals and infectious diseases experts with the objective to portray a textbook case of acute viral bronchitis. In their opening statement, SPs described their main complaint (“*I have been coughing for a few days”*), and immediate medical history (“*I had a cold last week, but now it’s better*”). The statement was rehearsed multiple times during the training so that all patients would reproduce it word for word every time. If the standardized patient was supposed to play the role of the reticent patient, s-he would add: “*I don’t want antibiotics, unless this is really necessary*”. The wording of this statement was based on preliminary formative work during which public and private doctors shared their experience and perceptions that patients would often “come to obtain antibiotics”.

Following this opening statement, fieldworkers were trained to answer any question that a doctor could ask according to a pre-determined script. A long list of possible questions and their answers, which formed the basis of the training, is provided in the table below.

Lagarde M, Blaauw D. *BMJ Glob Health* 2023; 8:e012374. doi: 10.1136/bmjgh-2023-012374

Appendix Table A1. Standardized responses to doctors' potential questions

Question	Response
When did you have the cold?	At the beginning of last week.
What was wrong? How was that cold?	It was like a normal cold. I had a blocked nose, a sore throat and my nose was running. I had a bit of headache. I felt quite tired/I had no energy. And I was coughing. **NOT:** shivering ; dizziness, body pain
How long did the cold last?	4-5 days
Did you take anything for the cold? / did you see a doctor?	I only took Panado/disprin and Medlemon / Stoney And for the cold I've been taking cough syrup / Benylin. I started feeling better after 4-5 days. But the cough hasn't stopped.
Is anything coming up when you cough?	No
Is your cough dry?	Yes
What you are coughing up – what does it look like?	Nothing really comes up. Some white mucus sometimes. **NOT:** yellow or green-ish or blood
It is not green or yellow.	No
Is there any blood?	No
When are you coughing?	Throughout the day. It really bothers me.
Do you have/ have you had a fever?	No
Do/did you have any earache?	No
Is your throat sore now?	No it's not sore when I swallow.
Is the cough worse at night? Or worse in the morning?	No but it will sometimes keep me up at night - I cough more when I lie down.
Have you had this before?	I have had colds before sometimes with coughing. But this coughing seems a bit worse than before.
Does anything make the coughing better / help the coughing?	I have been taking cough syrup. It doesn't help really.
Do you have any chest pain? Are you in pain?	No
Is it painful when you breathe?	No
Do you feel short of breath/difficulty breathing?	No.
Do you have difficulty walking up the stairs or up a hill?	No.
Do you have any shortness of breath at night?	No
Have you had any wheezing/ whistling noise when you breathe?	No. If asked about "wheezing" – ask clarification: "What is that?"
Is anyone else in your family sick?	My flatmate / friend had the flu just before me. They are fine now.
Did you have any breathing problems as a child?	I don't think so. I've never heard that I did.

**Question**	**Response**
Have you ever had pneumonia?	I don’t know. I don’t think so.
Have you ever had TB?	No
Have you had any other lung problems before?	No
Have you had any nausea or vomiting?	No
Have you had any diarrhoea?	No
Are you allergic to anything?	No, not that I know of.
Are you having night sweats?	No
Sweating a lot at night?	No
Are you losing weight? Have you lost weight recently?	No
Do you smoke? Have you ever smoked?	No
Are you HIV positive?	No
When did you last check for HIV?	About 6 months ago / at the beginning of the year
Where/how were you last tested?	Either one of: (i) I wanted to give blood ; (ii) There was a campaign on campus; (iii) I broke up with my boyfriend when I found out he was cheating so I got tested
Do you have any other medical problem (diabetes? Hypertension? Asthma? Pneumonia?)	No
Are you taking any other medication?	No
Do you drink?	Sometimes, with friends. OR never

2.2. Training of standardised patients

The training of standardised patients worked through three key elements: learning the clinical case (medical history, presenting symptoms, patient's attitude, responding to the provider's questions) ; navigating the healthcare system (what to expect in the public and private clinics, accessing study doctors, how to avoid invasive examinations) and completing the standardised checklist and post-consultation questionnaire (preparing before the consultation, capturing time before and after the consultation, recognising doctor's physical examinations).

Over the 10 days of the training, individuals gradually learnt and rehearsed the different elements of the standardised patient case. In addition to usual role-plays and collective training exercises to get familiar with the material and expectations, they practised through numerous mock consultations with the training facilitators, other training doctors, and then with real confederate doctors in private practice, and in one public facility for a pilot round. Feedback given to SPs included believability, consistency, and quality of recall. As a further guarantee of quality and validity, we dropped two individuals who were unable to meet the expected quality standards.

2.3. Differential diagnosis

Together with the location (urban South Africa) and time (winter) of the consultation, the persistent cough is potentially consistent with a number of illnesses: tuberculosis, pneumonia, acute bronchitis (bacterial or viral), asthma, allergic rhinitis (see Appendix Table 2 below for details about each alternative diagnosis and reasons for ruling it out).

Appropriate questioning and examination of the patient by the provider would uncover that the cough is productive and brings up clear mucus, but other than that the patient does *not* present any symptom consistent with the most likely alternative ailments: the patient has not had any fever; their sputum is not yellow-green (both symptoms would provide a reason to suspect some bacterial infection; their absence rules out bacterial bronchitis), nor does it contain blood (suggestive of tuberculosis); the patient has not experienced any shortness of breath and has a clear chest on examination (ruling out pneumonia); asthma can be ruled out by the absence of wheezing on exhalation (either reported by the patient or checked through auscultation), or broncho-obstruction measured by a peak expiratory flow; and the problem is a once-off episode following a recent cold (which, together with the lack of a history of allergies, rules out allergic concerns). Furthermore, the patient is young and generally healthy with no co-morbidities, which should further alleviate doctors' potential concerns of complications in immune-suppressed or susceptible individuals such as children or the elderly, which often fuel inappropriate antibiotic prescribing.

Lagarde M, Blaauw D. *BMJ Glob Health* 2023; 8:e012374. doi: 10.1136/bmjgh-2023-012374

Appendix Table A2. Alternative diagnoses for the case presented

Diagnosis	Typical presentation	Recommended patient management	Symptoms (or lack thereof) that make the diagnosis less likely in the SP case
Tuberculosis	Persistent cough (more than 2 weeks), productive (may have blood), weight loss, night sweats.	Refer to specialist centre to confirm diagnosis with a sputum test. Initiate treatment when diagnosis confirmed.	No blood in sputum; no weight loss; no night sweats; cough less than 2 weeks.
Pneumonia	Persistent cough, productive, fever, crackles on chest auscultation, shortness of breath.	Antibiotics. Severe cases may require hospitalisation for intravenous antibiotics.	No fever; no breathlessness; clear chest (no crackling sound).
Asthma	Episodes of cough, tight chest, wheezing, difficulty breathing.	Inhalers (bronchodilators, steroids), or tablets if more severe (Leukotriene receptor antagonists, theophylline, steroids).	No breathlessness or wheezing during the episode described; no history of asthma or allergies, normal peak expiratory flow.
Bacterial bronchitis	Cough, productive (may be yellow/green), shortness of breath, fever.	Antibiotics not recommended, except for pertussis (whooping cough).	Absence of fever; clear mucus.
Allergic rhinitis	Seasonal runny nose, itchy eyes, sneezing, occasional cough.	Anti-histamines.	No particular trigger; constant cough; no history of allergies, no conjunctivitis.
Viral bronchitis	Persistent cough, productive (usually clear mucus).	Relieve symptoms (analgesics cough suppressants). Antibiotics not recommended.	NA

Lagarde M, Blaauw D. *BMJ Glob Health* 2023; 8:e012374. doi: 10.1136/bmjgh-2023-012374

2.4. Checklist

The table below shows the full list of items related to the consultation filled by fieldworkers in a questionnaire immediately after the consultation. The list was established with doctors and medical experts to cover the essential and recommended questions and examinations that would be done in a thorough consultation.

Appendix Table A3. Post-consultation checklist

**Panel A: History-taking checklist**	
	Did the doctor ask you…
1.	how long have you been coughing for
2.	have you had fever or high temperature
3.	if anything came up when you coughed or if it was dry
4.	whether you have been coughing up blood
5.	if you have had a similar problem before
6.	if you have recently lost weight
7.	if you have been sweating at night
8.	if you have chest pain
9.	If you have difficulty breathing / are short of breath
10.	if you are making a whistling noise when you cough
11.	about childhood illnesses especially re: cough or breathing problems
12.	if you have a sore throat
13.	if you have earache
14.	more details about the cold
15.	if you have any allergies or hay fever
16.	if you smoke
17.	about your occupation
18.	if you are on any medication
19.	If you have had TB before
20.	about your HIV status
**Panel B: Physical examination checklist**	
	When s-he examined you, did the doctor…
1.	take your temperature
2.	take your blood pressure
3.	take your pulse
4.	examine your throat
5.	palpate for lymph nodes (below throat and ears)
6.	examine your ears with ear set (otoscope)
7.	listen to your heart (centre of your chest)
8.	listen to your lungs (back and/or front)
9.	tap your lungs (percuss)
10.	test your peak expiratory flow
11.	test your oxygen saturation

3. Construction of variables

3.1. Consultation characteristics (SP data)

In the analysis of the predictors of antibiotic prescribing, we included variables that known to be behavioural predictors over-prescribing or potentially amenable to policy intervention.

Based solely on the standardised-patients data, we constructed the following variables:

- **Longer consultation**: since standardised patients were trained to record the time of the start and of the consultation, we were able to precisely estimate the duration of all consultations. Pooling data from all consultations, we defined as "longer consultation" any consultation longer than the median duration of 8 minutes.
- **Retrieving key information**: following discussions with infectious diseases and primary care experts, we determined that three elements of information were critical to rule out the need for antibiotics for the patient: (1) the absence of fever (this information was considered as known by providers if they either asked the patient or took the temperature directly); (2) a clear throat (which would be ascertained by the provider if they examined the patient's throat) and (3) clear lungs (evident if a provider listened to the patient's lungs). We constructed a binary variable taking the value 1 when a provider had gathered all three elements of information, based on the data recorded by the standardised patient.
- **Consultation occurred late in the day**: following evidence from psychology, studies in high-income settings have shown that providers are more prone to using automatic behaviours in consultations occurring later in the working day, as fatigue is greater. Given differences in the way consultations are organised in the public and private sectors,[4] a "late" consultation was defined as one occurring after 10:15am in the public sector (the median start time for a consultation in our data) and after 1:58pm in the private sector (the median start time for a private consultation in our data).

3.2. Provider characteristics (interview data)

Using data from providers' interview data, we constructed the following variables:

- **High AMR knowledge**: the interview included a five-question quiz aiming to assess individual providers' knowledge of antimicrobial resistance. We computed an index of knowledge by summing the number of correct answers to the five questions (see questions in Box 1 below). We constructed the variable "high AMR knowledge" taking the value 1 if a provider had scored 4 or 5 out of 5.
- **Knows case is viral**: during the interview, providers were shown several clinical vignettes briefly describing the case of patients. One was depicting the same clinical case presented by the standardised patient.[5] At the end of the case providers were asked whether they thought the most likely cause of the patient's illness was a virus or a bacteria (the questionnaire also allowed for "I don't know"). The variable takes the value 1 for individuals who responded that the most likely cause was a virus.
- **Believes AB will not help recover**: using the same clinical vignette, providers were later asked to evaluate "if the patient was given antibiotics what is the probability that they would recover *more*

[4] In the public sector, most consultations occur in the morning, and rarely after the start of the afternoon. By contrast, it is not uncommon for private practices to be closed in the morning, or open later than public clinics, and welcome patients late in the afternoon.

[5] The case description in the vignette was: "a 25 year old man reports having a sore throat and rhinorrhoea for 10 days. However, over the last 5 days he has also developed a persistent cough throughout the day. The cough is productive of a white mucoid phlegm. On examination he was found to have a temperature of 37.1ºC, a respiratory rate of 17 breaths per minute, a clear throat and clear lungs."

quickly than if they were *not* given antibiotics”. We constructed a binary variable taking the value 1 if providers said the likelihood was equal or less than 40%.

- **Thinks the patient is unlikely to come back if not given antibiotics:** using the same clinical vignette of the SP case, providers were asked to evaluate “if the patient is not given antibiotics, what is the probability that they will not come back?”. We constructed a binary variable taking the value 1 if providers said the likelihood was equal or more than 60%.

Box 1: AMR quiz

1. What is antibiotic resistance?

A. When drugs don’t work because they are past their use-by date
B. When patients’ bodies become immune to antibiotics
C. When antibiotics no longer kill resistant bacteria as well as they used to (correct)
D. When a doctor won’t give patient antibiotics even if a patient is ill

2. What can cause antibiotic resistance?

A. It happens naturally over time
B. It happens when patients take antibiotics even though they don’t have a bacterial infection
C. It happens when patients take the wrong antibiotics for an infection
D. All of the above (correct)

3. True or False? If someone has not taken many courses of antibiotics in their lives, they won’t be affected by the resistance

A. True
B. False (correct)

4. When was the last time a major new class of antibiotics was introduced?

A. Last year
B. 5 years ago
C. 15 years ago
D. 30 years ago (correct)

5. Which of these treatments would become dangerous if antibiotics became ineffective?

A. Transplants
B. Chemotherapy
C. Caesarean sections
D. All of them (correct)

4. Additional results

Appendix Figure A3: Choice of type of antibiotics, by sector and type of private doctor

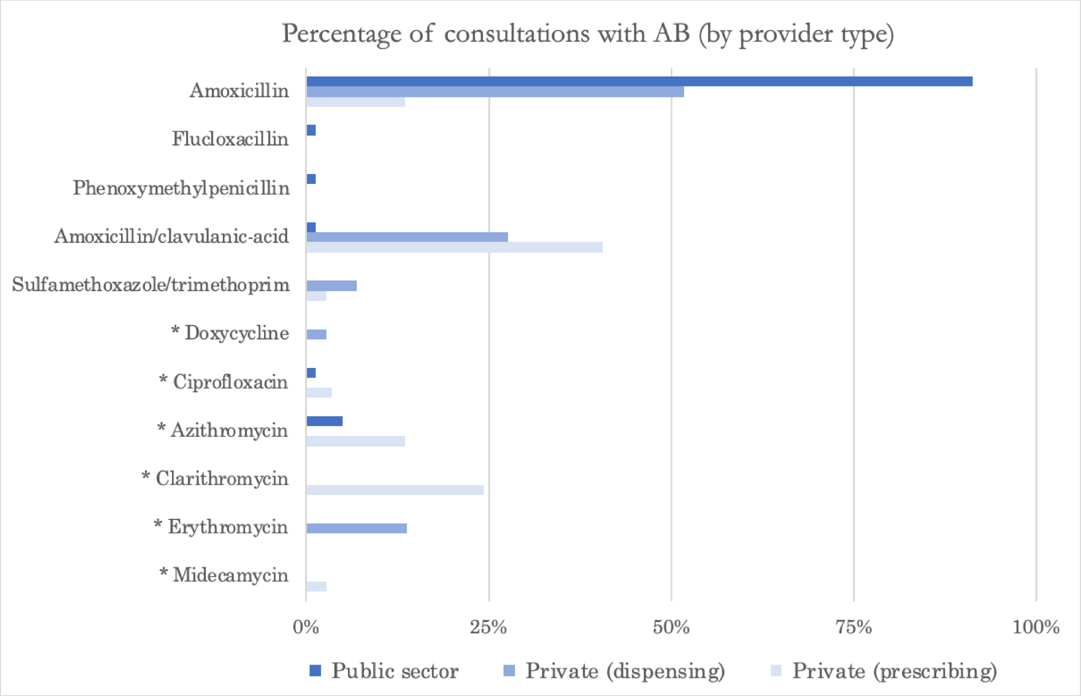

Notes: The frequency with which each listed active ingredient was contained in antibiotics prescribed or dispensed to standardised patients, for each sector. Active ingredients indicated by an asterisk are on the WATCH list of the WHO, due to resistance and toxicity concerns.

Appendix Table A4. Correlates of antibiotic prescribing, whole sample

	OR	p-value
Private sector	0.60 (0.25-1.47)	0.267
Longer consultation	0.41 (0.21-0.79)	0.008
Provider retrieved key information	0.85 (0.37-1.93)	0.689
Consultation late in the day	1.62 (0.84-3.13)	0.147
Patients waiting	0.99 (0.95-1.03)	0.495
Observations	201	

Notes: Estimates are from a logit model, performed on all interactions between standardized patients and providers.

Appendix Table A5. Knowledge of providers – sensitivity analysis

	All providers	Public providers	Private providers	pval[a]
**Panel A: inputting low knowledge**				
Knows cause of illness is viral	0.58 (0.49)	0.41 (0.50)	0.72 (0.45)	<0.001
Thinks AB unlikely to help patient recover	0.41 (0.49)	0.21 (0.41)	0.56 (0.50)	<0.001
Believes patient won't come back if no antibiotics	0.69 (0.46)	0.85 (0.36)	0.57 (0.50)	<0.001
AMR knowledge score (out of 5)	2.12 (1.74)	1.23 (1.43)	2.84 (1.63)	<0.001
**Panel B: inputting high knowledge**				
Knows cause of illness is viral	0.89 (0.32)	0.88 (0.33)	0.90 (0.30)	0.321
Thinks AB unlikely to help patient recover	0.71 (0.45)	0.68 (0.47)	0.74 (0.44)	0.596
Believes patient won't come back if no antibiotics	0.39 (0.49)	0.39 (0.49)	0.39 (0.49)	0.339
AMR knowledge score (out of 5)	3.65 (1.36)	3.54 (1.61)	3.74 (1.11)	0.973
Number of observations	180	80	100	

Notes: Numbers show mean or proportion with standard deviations in parentheses. AMR=Antimicrobial Resistance; AB=antibiotics. For the facility sample data on average waiting time are based on information collected by SPs. For all providers who did not take part in the follow-up interview, we inputted the lowest possible level of knowledge in Panel A: providers ignoring that the cause of illness is viral, thinking that AB are likely to help the patient recover, believing that the patient would not come back if not given AB, and with a score of 0 out of 5 for AMR. In panel B, we inputted the highest level of knowledge: providers knowing that the cause of illness is viral, thinking that AB are unlikely to help the patient recover, not believing that the patient would not come back if not given AB, and with a perfect score of 5 out of 5 for AMR.
[a] The p-value is based on t-test for means and Chi-square tests for proportions comparing the private and public sector characteristics.

Lagarde M, Blaauw D. *BMJ Glob Health* 2023; 8:e012374. doi: 10.1136/bmjgh-2023-012374

Appendix Table A6. Correlates of antibiotic prescribing, public sector

	(1) Full sample, parsimonious model		(2) Interview sample, parsimonious model		(3) Interview sample, full model	
	OR	p-value	OR	p-value	OR	p-value
Longer consultation	0.89 (0.33-2.42)	0.817	0.50 (0.13-1.94)	0.315	0.48 (0.11-2.05)	0.323
Provider retrieved key information	0.46 (0.08-2.78)	0.397	0.44 (0.03-6.15)	0.542	0.41 (0.03-6.23)	0.521
Consultation late in the day	0.84 (0.31-2.24)	0.724	1.41 (0.33-6.09)	0.643	1.43 (0.32-6.46)	0.640
Patients waiting	0.98 (0.94-1.03)	0.444	0.98 (0.92-1.04)	0.575	0.98 (0.92-1.05)	0.627
High AMR knowledge					1.58 (0.25-9.96)	0.627
Knows case is viral					0.77 (0.12-5.00)	0.782
Believes AB will not help recover					1.09 (0.22-5.27)	0.917
Believes patient won’t come back if no AB					1.35 (0.31-5.86)	0.692
Observations	102		58		58	

Notes: Estimates are from a logit model, performed on all interactions between standardized patients and providers. In column 1, data include all interactions in the public sector. In columns 2 and 3, data include all interactions between SPs and providers who agreed to take part in the follow-up interview.

Appendix Table A7. Correlates of antibiotic prescribing, private sector

	(1) **Full sample, parsimonious model**		(2) **Interview sample, parsimonious model**		(3) **Interview sample, full model**	
	OR	p-value	OR	p-value	OR	p-value
Longer consultation	0.20 (0.07-0.58)	0.003	0.16 (0.05-0.55)	0.003	0.15 (0.04-0.55)	0.004
Provider retrieved key information	1.29 (0.47-3.49)	0.621	1.40 (0.47-4.16)	0.543	1.38 (0.43-4.41)	0.585
Consultation late in the day	3.21 (1.22-8.45)	0.018	3.60 (1.24-10.43)	0.018	3.69 (1.18-11.60)	0.025
Patients waiting	1.01 (0.88-1.15)	0.902	0.96 (0.82-1.13)	0.647	0.96 (0.81-1.15)	0.687
GP is dispensing	2.06 (0.75-5.67)	0.160	2.00 (0.65-6.15)	0.225	1.54 (0.45-5.34)	0.494
High AMR knowledge					0.36 (0.11-1.16)	0.088
Knows case is viral					0.17 (0.02-1.57)	0.117
Believes AB will not help recover					0.85 (0.21-3.42)	0.815
Believes patient won't come back if no AB					0.89 (0.26-3.08)	0.857
Observations	99		81		81	

Notes: Estimates are from a logit model, performed on all interactions between standardized patients and providers. In column 1, data include all interactions in the public sector. In columns 2 and 3, data include all interactions between SPs and providers who agreed to take part in the follow-up interview.

Lagarde M, Blaauw D. *BMJ Glob Health* 2023; 8:e012374. doi: 10.1136/bmjgh-2023-012374

5. Strobe Statement

	Item No	Recommendation	Page No
**Title and abstract**	1	(*a*) Indicate the study's design with a commonly used term in the title or the abstract	abstract
		(*b*) Provide in the abstract an informative and balanced summary of what was done and what was found	abstract
**Introduction**			
Background rationale	2	Explain the scientific background and rationale for the investigation being reported	p. 3-5
Objectives	3	State specific objectives, including any prespecified hypotheses	p. 5
**Methods**			
Study design	4	Present key elements of study design early in the paper	p. 8-9
Setting	5	Describe the setting, locations, and relevant dates, including periods of recruitment, exposure, follow-up, and data collection	p. 6-7
Participants	6	(*a*) Give the eligibility criteria, and the sources and methods of selection of participants	p. 11
Variables	7	Clearly define all outcomes, exposures, predictors, potential confounders, and effect modifiers. Give diagnostic criteria, if applicable	p. 12-13
Data sources/ measurement	8	For each variable of interest, give sources of data and details of methods of assessment (measurement). Describe comparability of assessment methods if there is more than one group	p. 12-13
Bias	9	Describe any efforts to address potential sources of bias	NA
Study size	10	Explain how the study size was arrived at	p. 11
Quantitative variables	11	Explain how quantitative variables were handled in the analyses. If applicable, describe which groupings were chosen and why	p. 12-13
Statistical methods	12	(*a*) Describe all statistical methods, including those used to control for confounding	p. 12-13
		(*b*) Describe any methods used to examine subgroups and interactions	NA
		(*c*) Explain how missing data were addressed	NA
		(*d*) If applicable, describe analytical methods taking account of sampling strategy	NA
		(*e*) Describe any sensitivity analyses	NA
**Results**			
Participants	13	(a) Report numbers of individuals at each stage of study—eg numbers potentially eligible, examined for eligibility, confirmed eligible, included in the study, completing follow-up, and analysed	Appendix Figures 2 and 3
		(b) Give reasons for non-participation at each stage	
		(c) Consider use of a flow diagram	
Descriptive data	14	(a) Give characteristics of study participants (eg demographic, clinical, social) and information on exposures and potential confounders	Table 2
		(b) Indicate number of participants with missing data for each variable of interest	Tables 1-2

Outcome data	15	Report numbers of outcome events or summary measures	Table 2
Main results	16	(*a*) Give unadjusted estimates and, if applicable, confounder-adjusted estimates and their precision (eg, 95% confidence interval). Make clear which confounders were adjusted for and why they were included	p. Table 2, Appendix Tables,
		(*b*) Report category boundaries when continuous variables were categorized	Appendix Table
		(*c*) If relevant, consider translating estimates of relative risk into absolute risk for a meaningful time period	NA
Other analyses	17	Report other analyses done—eg analyses of subgroups and interactions, and sensitivity analyses	p. 16
**Discussion**			
Key results	18	Summarise key results with reference to study objectives	p. 19-20
Limitations	19	Discuss limitations of the study, taking into account sources of potential bias or imprecision. Discuss both direction and magnitude of any potential bias	p. 20-21
Interpretation	20	Give a cautious overall interpretation of results considering objectives, limitations, multiplicity of analyses, results from similar studies, and other relevant evidence	p. 21-22
Generalisability	21	Discuss the generalisability (external validity) of the study results	p. 23
**Other information**			
Funding	22	Give the source of funding and the role of the funders for the present study and, if applicable, for the original study on which the present article is based	Acknowledgem ent

Note: An Explanation and Elaboration article discusses each checklist item and gives methodological background and published examples of transparent reporting. The STROBE checklist is best used in conjunction with this article (freely available on the Web sites of PLoS Medicine at http://www.plosmedicine.org/, Annals of Internal Medicine at http://www.annals.org/, and Epidemiology at http://www.epidem.com/). Information on the STROBE Initiative is available at www.strobe-statement.org.

6. Reflexivity Statement

As authors submitting research from international partnerships between high-income countries and low- and/or middle-income countries, the journal requires to include a reflexivity statement, which includes the following 15 questions.

1. How does this study address local research and policy priorities?

This study directly addresses a policy and research priority in South Africa. South Africa has very high rates of anti-microbial resistance (AMR) and has developed a range of national initiatives to address this issue. This study highlights the contribution of antibiotic misuse in primary care in South Africa to the problem.

2. How were local researchers involved in study design?

This study was an equal partnership between the United Kingdom and South African researchers at all stages. Both ML and DB were involved in the development of the proposal, planning of the study and execution of the project. This was facilitated through frequent communication and study design workshops held between ML and DB in South Africa.

3. How has funding been used to support the local research team?

Approximately 60% of the total project budget was directly allocated to the South African research team. These funds supported researcher time, local institutional support, and fieldwork costs.

4. How are research staff who conducted data collection acknowledged?

Both ML and DB who oversaw the project are included as authors. Most data collection was outsourced to a local fieldwork company who are acknowledged in the paper for their contribution.

5. Do all members of the research partnership have access to study data?

Yes. All members of the partnership have access to data.

6. How was data used to develop analytical skills within the partnership?

Both ML and DB are experienced analysts. The main project analysis was shared between both of them, each taking the lead on different aspects. The final analyses of this paper were mostly done by ML, as the first author.

7. How have research partners collaborated in interpreting study data?

Local research partners have directly contributed to data analysis and the interpretation of the results. This was done through joint analysis workshops, preparation of the results for presentation together, and joint writing workshops.

8. How were research partners supported to develop writing skills?

The research team writing this paper is composed of senior academics. Writing tasks were shared among the team.

9. How will research products be shared to address local needs?

The main findings presented in this paper have been shared with local policymakers and decision-makers through direct meetings, feedback workshops and through policy briefs distributed locally.

10. How is the leadership, contribution and ownership of this work by LMIC researchers recognised within the authorship?

DB is recognised as a joint author of this work.

11. **How have early career researchers across the partnership been included within the authorship team?**

This paper was the work of ML and DB. There are no other early career researchers included in the authorship team.

12. How has gender balance been addressed within the authorship?

One author is female (ML) and one author is male (DB). ML is the first author of this paper.

13. How has the project contributed to training of LMIC researchers?

The authorship team for this paper is composed of senior researchers.

14. How has the project contributed to improvements in local infrastructure?

Part of the South African funding was used for local institutional support, primarily for institutional overheads and administrative staff.

15. What safeguarding procedures were used to protect local study participants and researchers?

Ethical research procedures were maintained throughout, including consent for all data collection and ensuring confidentiality. The study proposal was approved jointly by ethics committees in the United Kingdom and South Africa. Approval for the research in public facilities was also obtained from the appropriate national and local research committees.
